# Assessment of Greenhouse Tomato Anthesis Rate Through Metabolomics Using LASSO Regularized Linear Regression Model

**DOI:** 10.3389/fmolb.2022.839051

**Published:** 2022-03-01

**Authors:** Ratklao Siriwach, Jun Matsuzaki, Takeshi Saito, Hiroshi Nishimura, Masahide Isozaki, Yosuke Isoyama, Muneo Sato, Masanori Arita, Shotaro Akaho, Tadahisa Higashide, Kentaro Yano, Masami Yokota Hirai

**Affiliations:** ^1^ RIKEN Center for Sustainable Resource Science, Yokohama, Japan; ^2^ Institute of Vegetable and Floriculture Science, NARO, Tsukuba, Japan; ^3^ Mie Prefecture Agricultural Research Institute, Matsusaka, Japan; ^4^ National Institute of Genetics, Mishima, Japan; ^5^ National Institute of Advanced Industrial Science and Technology, Tsukuba, Japan; ^6^ Bioinformatics Laboratory, Department of Life Sciences, School of Agriculture, Meiji University, Kawasaki, Japan

**Keywords:** metabolome, metabolites, tomato, anthesis rate, machine learning, LASSO, trigonelline

## Abstract

While the high year-round production of tomatoes has been facilitated by solar greenhouse cultivation, these yields readily fluctuate in response to changing environmental conditions. Mathematic modeling has been applied to forecast phenotypes of tomatoes using environmental measurements (e.g., temperature) as indirect parameters. In this study, metabolome data, as direct parameters reflecting plant internal status, were used to construct a predictive model of the anthesis rate of greenhouse tomatoes. Metabolome data were obtained from tomato leaves and used as variables for linear regression with the least absolute shrinkage and selection operator (LASSO) for prediction. The constructed model accurately predicted the anthesis rate, with an R^2^ value of 0.85. Twenty-nine of the 161 metabolites were selected as candidate markers. The selected metabolites were further validated for their association with anthesis rates using the different metabolome datasets. To assess the importance of the selected metabolites in cultivation, the relationships between the metabolites and cultivation conditions were analyzed *via* correspondence analysis. Trigonelline, whose content did not exhibit a diurnal rhythm, displayed major contributions to the cultivation, and is thus a potential metabolic marker for predicting the anthesis rate. This study demonstrates that machine learning can be applied to metabolome data to identify metabolites indicative of agricultural traits.

## 1 Introduction

Tomatoes (*Solanum lycopersicum* L.) are produced worldwide, with the highest rates of production among non-grain crops after potatoes (FAOSTAT, 2018). The high year-round production of tomato fruits has been facilitated by greenhouse cultivation in many countries. Greenhouse cultivation provides the optimal environmental conditions, such as temperature, humidity, and light conditions, needed to grow plants (Peet and Welles, 2005). However, in addition to the automatic control of environmental conditions, prompt treatment by tomato growers is necessary to mitigate the effects of extreme weather conditions. For example, extreme heat causes pre-harvest physiological disorders, resulting in fruit cracking and blossom drop in tomato plants. For such extreme heat, temporary equipment and/or manual control is required to lower the temperature in the greenhouse (Liebisch et al., 2009; Saure, 2014). Therefore, for greenhouse cultivation, there is a need to continuously and adequately manage the environmental conditions inside greenhouses. Moreover, the morphological or physiological status of tomato plants can be used to infer subsequent plant growth and outcome (crop harvest). This means that more favorable growth conditions could be investigated and elucidated to enhance plant growth and maximize tomato fruit production. At present, tomato growers empirically control the growth conditions in greenhouses according to extreme weather conditions and plant vigor.

Recently, omics data have been utilized in phenotype prediction and the identification of genes that control traits of interest. Among the omics data, gene expression data have been employed, as gene expression profiles can be easily collected by microarray experiments or sequencing technologies (Yamamoto et al., 2016; Gao et al., 2018; Liabeuf et al., 2018). Yano et al. (2006) introduced an accurate prediction method for phenotypes with comprehensive gene expression profiles using a model on a statistical index and correspondence analysis (CA). In addition to transcriptome analysis, comprehensive metabolite profiles (patterns of metabolite contents across a wide range of experimental conditions) have also become practical with high-throughput mass spectrometry-based technologies. Since metabolites are directly related to phenotypes rather than events of gene expression, phenotype prediction using metabolome data is a promising strategy with which to considerably improve predictability.

There are both direct and indirect approaches to the omics analysis of a target trait. Omics data (e.g., gene expression and/or metabolic profiles) obtained from a given organ represent the genetic and physiological status of the same organ. Therefore, omics data are directly available to identify genes and/or metabolites controlling a given trait in an organ. For example, omics data from the fruit of tomato plants rather than other organs (e.g., leaves) are suitable for the detection of genes and metabolites that play a key role in fruit development. However, the direct approach is unfavorable because for the collection of omics data, fruits need to be removed from the plant. To maximize the quantity of fruit production in the greenhouse, it is better to use vegetative organs, such as, rather of the fruit, for the collection of omics data. If omics data from vegetative organs is able to accurately represent the status of tomato fruit, the indirect approach could also prove to be effective and efficient for the identification of genes and metabolites for a trait, as well as for phenotype prediction.

The metabolic profiling of vegetative organs has been reported to be highly correlated with the quantity of tomato fruit produced. For example, the association between vegetative and reproductive growth of greenhouse tomatoes has been studied for a long time (Khan and Sagar, 1969; Tanaka and Fujita, 1974). The allocation of assimilated carbon between vegetative organs (leaves) and reproductive organs (flowers and fruits) is controlled by genetic and environmental factors, such as light intensity and temperature (Dinar and Rudich, 1985; Heuvelink and Buiskool, 1995). Previous studies have also suggested that the metabolic profiles of vegetative organs, rather than reproductive organs, are attractive and suitable for the construction of a prediction model for fruit yield.

When the metabolic profiles in a vegetative organ are effective in accurately predicting fruit yield, the profiles of a metabolite(s) must be strongly associated with yield. The metabolite(s) allows us to predict not only the yield, but also the traits that are highly correlated with the yield. For example, the effective number of flowers that eventually develop mature fruits is correlated with the yield. This suggests that the effective number of flowers newly generated within a period (e.g., a week) in the greenhouse, referred to as the “anthesis rate” in this study, is an effective index for the prediction of fruit production. In addition, this index has practical and diagnostic advantages for maximizing fruit production. When the predicted anthesis rate is too low for commercial fruit production, the environmental condition can be reconsidered to increase the rate. The improvement enhances the subsequent plant growth and increases the effective number of flowers, then maximizes tomato fruit production.

In this study, we present a statistical model with comprehensive metabolic profiles aimed at maximizing tomato fruit production in greenhouses, wherein the metabolic profiles in leaves were employed to predict the anthesis rate. Because metabolome data is a high-dimensional multivariate data, variable selection is a crucial step to characterize the underlying patterns of these variables and narrow them down to find significant variables. Sparse modeling including the least absolute shrinkage and selection operator (LASSO) model that we applied in this study is widely used in various areas of data-driven science (Rasmussen and Bro, 2012; Rish and Grabarnik, 2014). LASSO model has the ability to perform variable selection by reducing the number of variables. In the LASSO model, significantly contributing variables are weighted with large coefficients, while non-contributing variables are weighted with zero or near-zero coefficients. Consequently, we also identified metabolites that strongly contributed to the prediction of the anthesis rate. To date, the control of the environmental conditions in greenhouses has mainly relied on the experience and knowledge of experts in tomato fruit production. However, the use of machine learning and multivariate analysis with comprehensive metabolic profiles in vegetative organs allows us to not only predict fruit production, but also to adjust the environmental conditions for the enhancement of tomato growth without a need for abundant practical experience. This novel strategy will provide innovative knowledge and skills in greenhouse cultivation for all tomato growers, as well as facilitate the economically efficient production of other crops under greenhouse conditions.

## 2 Materials and Methods

### 2.1 Plant Materials and Growth Conditions

Tomato plants were grown in greenhouses located in Tsukuba (36°2′4.88″ N, 140°6′2.9″ E) and Matsusaka (34°37′51.7″ N, 136°29′39.5″ E), Japan.

#### 2.1.1 Tsukuba Greenhouse (TK01)

In Tsukuba, in the experiment designated TK01, the seeds of the tomato cultivar Ringyoku (National Agricultural Research Organization, Tsukuba, Japan) and rootstock cultivar Maxifort (*S. lycopersicum* × *S. habrochaites*; De Ruiter Seeds, Bergschenhoek, Netherlands) were sown on 16 May 2016. CF Momotaro York (CFMY) seeds (Takii Seed, Kyoto, Japan) were sown on 23 May 2016. On day 14 after sowing (DAS), Ringyoku scions were grafted onto Maxifort rootstocks. On DAS 28 (13 June 2016), all seedlings were transplanted into rockwool blocks (Delta4, Grodan, Roermond, Netherlands) and placed on rockwool slabs (Grotop expert, Grodan) in a greenhouse with a plant density of 3.3 plants/m^2^. Culture liquid with an electrical conductivity (EC) of 3.4 mS/cm (15.8 me/L nitrate, 4.5 me/L P, 9.8 me/L K, 9.3 me/L Ca, 4.6 me/L Mg, 0.07 me/L Fe, 0.103 me/L B, 0.017 me/L Mn, 0.076 me/L Zn, 0.00120 me/L Cu, and 0.00083 me/L Mo) was administered *via* a drip. After 14 days of transplanting, culture liquid with an EC of 2.6 mS/cm was administered. To control the cultivation environment, a ubiquitous environment control system (Fujitsu, Kawasaki, Japan) was used. The greenhouse was ventilated during the day and heated overnight so that the daily mean temperature was maintained at 25°C. A heat pump (Green Package; Nepon, Tokyo, Japan) was operated from 20:00 to 04:00, with a target range of 16–20°C. The daytime relative humidity was controlled at 75% until 30 days after transplanting, and maintained at 70% thereafter. Nineteen days after transplanting, CO_2_ was added from 05:00 to 07:00 to reach a concentration of 800 ppm. Then, and until 105 days after transplanting (26 September 2016), CO_2_ was added to a concentration of 400 ppm all day.

#### 2.1.2 Matsusaka Greenhouse (IA04)

In Matsusaka, two sets of experiments (IA04 and IA06) were conducted. In the experiment designated IA04, the seeds of the tomato cultivars CFMY, C5-159 (Sakata Seed Co., Japan), C5-160 (Sakata Seed Co.), and C6-164 (Sakata Seed Co.) were sown on 27 July 2016. The seedlings grafted onto Maxifort rootstocks were transplanted on 1 September 2016. The plant density was set at 2.4 plants/m^2^ and then rearranged to be 3.6 plants/m^2^ in late January 2017. A rockwool culture system with drip fertigation was used in the greenhouse. The culture liquid was supplied with an EC of 3.0 mS/cm (16 me/L N, 4 me/L P, 8.0 me/L K, 8 me/L Ca, and 4 me/L Mg). The interior air temperature was controlled within the range of 13–27°C. The ideal humidity was 80%, and the CO_2_ concentration was 800 ppm normally without ventilation and 400 ppm with ventilation during cloudy weather.

#### 2.1.3 Matsusaka Greenhouse (IA06)

In another experiment, designated IA06, the seeds of the tomato cultivars CFMY, Ringyoku, and Managua (RIJK ZWAAN, Netherlands) were sown on 4 October 2016. The seedlings grafted onto Maxifort rootstocks were transplanted on 31 October 2016. The plant density was 2.4 plants/m^2^ in the first 3 months and then rearranged to 3.6 plants/m^2^. A rockwool culture system with drip fertigation was used in the greenhouse. The culture liquid was supplied with an EC of 3.0 mS/cm (16 me/L N, 4 me/L P, 8.0 me/L K, 8 me/L Ca, and 4 me/L Mg). The environmental conditions were controlled as in experiment IA04.

### 2.2 Measurement of Anthesis Rates

To measure the anthesis rates, we periodically counted the number of flowers that had not fallen off of each plant. The cumulative numbers of flowers (“cumulative anthesis”) were plotted (see Section 3 for details). From the cumulative anthesis plot, the anthesis rates were calculated from the gradients of a straight line between two neighboring time-points on the horizontal axis.

### 2.3 Metabolome Analysis

#### 2.3.1 Sampling of Tomato Leaves

In Tsukuba (TK01), the most basal leaflet of a fully developed and sunlit leaf was sampled for two replications every 2 h continuously for 24 h at one-week intervals for 4 weeks. A total of 192 leaf samples were collected from 16 August 2016 to 6 September 2016 (Ringyoku; *n* = 96, CFMY; *n* = 96). In Matsusaka, the fully developed upper leaves were sampled during 10:00–14:00 on 13 October 2016, and 19 January 2017, for IA04 for three replications, except for C5-160 for two replications (CFMY; *n* = 6, C5-159; *n* = 6, C5-160; *n* = 4, C6-164; *n* = 6) and on 19 January 2017 (6 replications) and 9 March 2017 (8 replicates) for IA06 (Ringyoku; *n* = 14, CFMY; *n* = 14, Managua; *n* = 14). The leaves were collected and flash-frozen in liquid nitrogen.

#### 2.3.2 Widely Targeted Metabolomic Analysis

The frozen leaf samples were freeze-dried and powdered. A small amount of samples (0.5–8.9 mg dry weight) was weighed and 1 ml/10 mg (TK01) or 4 mg (IA04 and IA06) dry weight of extraction solvent [80% (v/v) methanol and 0.1% (v/v) formic acid, with 8.4 nmol/L lidocaine and 210 nmol/L 10-camphorsulfonic acid as internal standards] was added. This mixture was shaken using a Shake Master Neo for 2 min at 1,000 rpm to extract the metabolites. After centrifugation for 1 min at 9,100 × g, the supernatant was diluted with the extraction solvent to obtain 0.4 mg/ml extracts. Next, 25 µL of the extract was dried, dissolved in 250 µL of ultra-pure water, and filtered using Millipore MultiScreenHTS384 well (Merck KGaA, Darmstadt, Germany). A 1-µL aliquot of this filtrate (0.04 mg/ml) was subjected to widely targeted metabolomics using liquid chromatography coupled with a tandem quadrupole mass spectrometer (LC-QqQ-MS) (UPLC coupled with Xevo TQ-S, Waters, Milford, MA, United States) (Sawada et al., 2009; Sawada et al., 2019). The analytical conditions are described in detail in Supplementary Tables S1–S3. The metabolome data were deposited in the DROP Met in PRIMe (the Platform for RIKEN Metabolomics) (DM0041, http://prime.psc.riken.jp/archives/data/DropMet/059/).

#### 2.3.3 Measurement of Relative Metabolite Contents

For the Tsukuba data (TK01), the peak areas of 501 target metabolites (including two internal standards) were processed as follows. Values below the detection limit were set to zero. The peak area of each metabolite in a leaf sample was divided by the mean peak area in the extraction solvent control from the same leaf sample to obtain the signal-to-noise ratio. In total, 161 metabolites were detected with signal-to-noise ratios above two in more than half of the leaf samples (Supplementary Table S3). The peak area of each metabolite was divided by that of the internal standard (lidocaine or 10-camphorsulfonic acid) to obtain the relative metabolite content.

The peak areas from the Matsusaka data (IA04 and IA06) were processed in the same manner as those from the Tsukuba data (TK01). After calculating the signal-to-noise ratio, the peak area of each metabolite was divided by that of the internal standard (lidocaine or 10-camphorsulfonic acid) to obtain the relative metabolite content.

### 2.4 Least Absolute Shrinkage and Selection Operator Regularized Linear Regression Model Analysis

LASSO regularization was used to extract essential metabolites to predict an anthesis rate. We constructed a prediction model of the anthesis rate using LASSO regularized linear regression analysis, called the LASSO model, to identify the “predictor metabolites” for the anthesis rate.

#### 2.4.1 Least Absolute Shrinkage and Selection Operator Model to Predict the Anthesis Rate in TK01

A LASSO model using metabolome data from TK01, named “M-model”, was constructed. Before training the model, the relative metabolite contents of each metabolite in all leaf samples were normalized to have a mean of zero and a standard deviation of one (that is, standardization). The LASSO model was implemented using sklearn.linear_model.Lasso in the Scikit-learn package (McKinney, 2010; Pedregosa et al., 2011).

The M-model was constructed by training the metabolic profiles of 161 metabolites from 192 leaf samples. The linear regression is expressed as:

$$y_{i}=\;w_{0}+w_{1}X_{i1}+\ldots +\;w_{m}X_{im},\;\;\;i\in \left[1,n\right],$$ (1)

where *y*_*i*_ is the anthesis rate of the plant with the *i*th leaf samples (1 ≤ *i* ≤ *n*, *n* = 192), *X*_*ij*_ is the relative metabolite content of the *j*th metabolite in the *i*th sample (1 ≤ *j* ≤ *m*, *m* = 161), *w*_*j*_ is the model coefficient of the *j*th metabolite (1 ≤ *j* ≤ *m*), and *w*_0_ is an intercept term. Here, *y*_*i*_ and *X*_*ij*_ are elements of a vector *y =* (*y*_1_, … , *y*_*n*_)^T^ and an *n × m* matrix *X*, respectively. The linear regression was trained with L1 regularization to perform both feature selection and regularization. The objective function to minimize is:

$$\underset{w}{\min}\frac{1}{2n}\left|\left|Xw-y|{|}_{2}^{2}+\alpha \right|\right|w|{|}_{1},$$ (2)

where $||Xw-y|{|}_{2}^{2}$ = $\sum_{i=1}^{n}{\left(X_{i}w-y_{i}\right)}^{2}$ is the sum of the squared errors, $||w|{|}_{1}$ = $\sum_{j=1}^{m}\left|w_{j}\right|$ is the L1-norm of the coefficient vector, and  α ≥0 is the penalty constant (Tibshirani, 1996). Thus, in the M-model, significantly contributing metabolites, called the selected metabolites, were weighted with large coefficients (either positive or negative), while non-contributing metabolites were weighted with zero coefficients. *R* ^2^ value of the M-model was calculated. The prediction accuracy was assessed by 10-fold cross-validation. *R* ^2^ value and the mean squared error (MSE) were used as accuracy metrics.

In addition, the second and third LASSO model training with environmental data (E-model) and combined metabolome and environmental data (C-model), respectively, were constructed in the same manner as the M-model. In the E-model, the *X* matrix contained only environmental factor data (solar irradiance, ambient temperature, relative humidity, and CO_2_ concentration). The *X* matrix in the C-model consisted of the metabolic profiles of 161 metabolites and environmental factor data.

#### 2.4.2 Least Absolute Shrinkage and Selection Operator Model for the Assessment of the Prediction Accuracy of the Predictor Metabolites

We also used the LASSO model to assess the ability and strength of the predictor metabolites in the M-model by expanding the metabolome data from different experimental designs. The predictor metabolites selected from the M-model were used to reconstruct the LASSO model with additional leaf samples from IA04 and IA06. The model was reconstructed in the same manner as the M-model by training the metabolic profiles of the predictor metabolites of 256 leaf samples from three greenhouses (TK01, IA04, and IA06).

### 2.5 Classification of Leaf Samples by Principal Component Analysis

The differences in leaf samples were evaluated by the PCA of their metabolic profiles. The relative metabolite content of each metabolite in all leaf samples was standardized. The PCA tool in the Scikit-learn package was used. The first two principal components of each leaf sample were used to project the leaf samples into a two-dimensional space. PCA was performed with two datasets, TK01 and a combined data of TK01, IA04, and IA06. For the PCA of TK01, the metabolic profiles of 161 metabolites from 192 leaf samples were used. For the PCA of data combined from TK01, IA04 and IA06, the metabolic profile of the predictor metabolites of 256 leaf samples from the three greenhouses (TK01, IA04, and IA06) were used.

### 2.6 Hierarchical Clustering Analysis of the Predictor Metabolites

To evaluate the similarities among the predictor metabolites, the metabolic profiles of 256 leaf samples from the three greenhouses (TK01, IA04, and IA06) were used for HCL. The Pearson correlation coefficient (*r*) of the relative metabolite contents for each pair of metabolites was calculated (Supplementary Figure S3 and Supplementary Table S4). Then, the distances between metabolites, namely, the “correlation distance” (1–*r*), were employed for agglomerative clustering. Linkage methods were applied to compute the distances between sub-clusters; then, a dendrogram was generated to mine metabolites showing similar profiles. The optimum linkage method was determined based on the cophenetic correlation coefficient. The best linkage method, which yielded the maximum cophenetic correlation coefficient, was used to create a hierarchical dendrogram (Jones et al., 2001). HCL was implemented using the Python library Scipy.

### 2.7 Network Analysis of the Predictor Metabolites With Correspondence Analysis

CA is a multivariate technique and is conceptually similar to PCA. In previous studies, CA has been used to clarify the associations between genes and experimental conditions in microarray analyses (Yano et al., 2006; de Tayrac et al., 2009). We employed CA for network analysis to discover the associations between the predictor metabolites and the associations between the predictor metabolites and the leaf sample characteristics, that is, experimental designs, cultivars, and sampling times.

CA was executed against metabolic profiles. The metabolome data were arranged in a matrix where the columns and rows correspond to the predictor metabolites selected by the M-model and 256 leaf samples from the three experimental designs, respectively. The relative metabolite contents of each metabolite in all leaf samples were standardized, and the minimum value was subtracted to prevent negative values. CA was performed using the FactoMineR library in R (Lê et al., 2008). Coordinates with *m-*1 dimensions were assigned to each metabolite and leaf sample, where *m* is the number of predictor metabolites. The coordinate values of all dimensions were retrieved (Supplementary Table S5).

#### 2.7.1 Network Analysis Between the Predictor Metabolites and the Leaf Sample Characteristics

The Euclidean distances for each pair of a metabolite and leaf sample were calculated using coordinates in all dimensions from CA. Theoretically, a smaller Euclidean distance indicates a higher association. Based on the histograms of the Euclidean distance (Supplementary Figure S4A), the 15th percentile of all distances was set as a threshold value to define a significant association. Pairs of a metabolite and leaf sample with distances less than the threshold were selected (Supplementary Table S6). The mean of the distances between each metabolite and each leaf sample characteristics were integrated to construct metabolic networks. Networks were constructed using py2cytoscape and NetworkX libraries in Python, and Cytoscape software (version 3.6.1) (Shannon et al., 2003; Hagberg et al., 2008; Ono et al., 2015). The associations between the metabolites were also evaluated in the same manner.

#### 2.7.2 Network Analysis Among the Predictor Metabolites

CA was used to determine the association among the predictor metabolites. The same process was performed to obtain pairwise Euclidean distances between the metabolites (Supplementary Tables S7, S8). The distances that passed the threshold were integrated to construct the metabolite networks.

### Statistical Analysis for the Anthesis Rates

In TK01, the significance of the anthesis rates between the cultivars was analyzed using the Mann-Whitney U test. The significance of the anthesis rates among the experimental designs (TK01, IA04, and IA06) was analyzed using the Kruskal–Wallis test with Conover’s multiple comparison test. Scipy in Python was used for the statistical analyses.

## 3 Results

### 3.1 Data Collection for Anthesis Rate, Leaf Metabolome, and Environmental Factors

**FIGURE 1:**
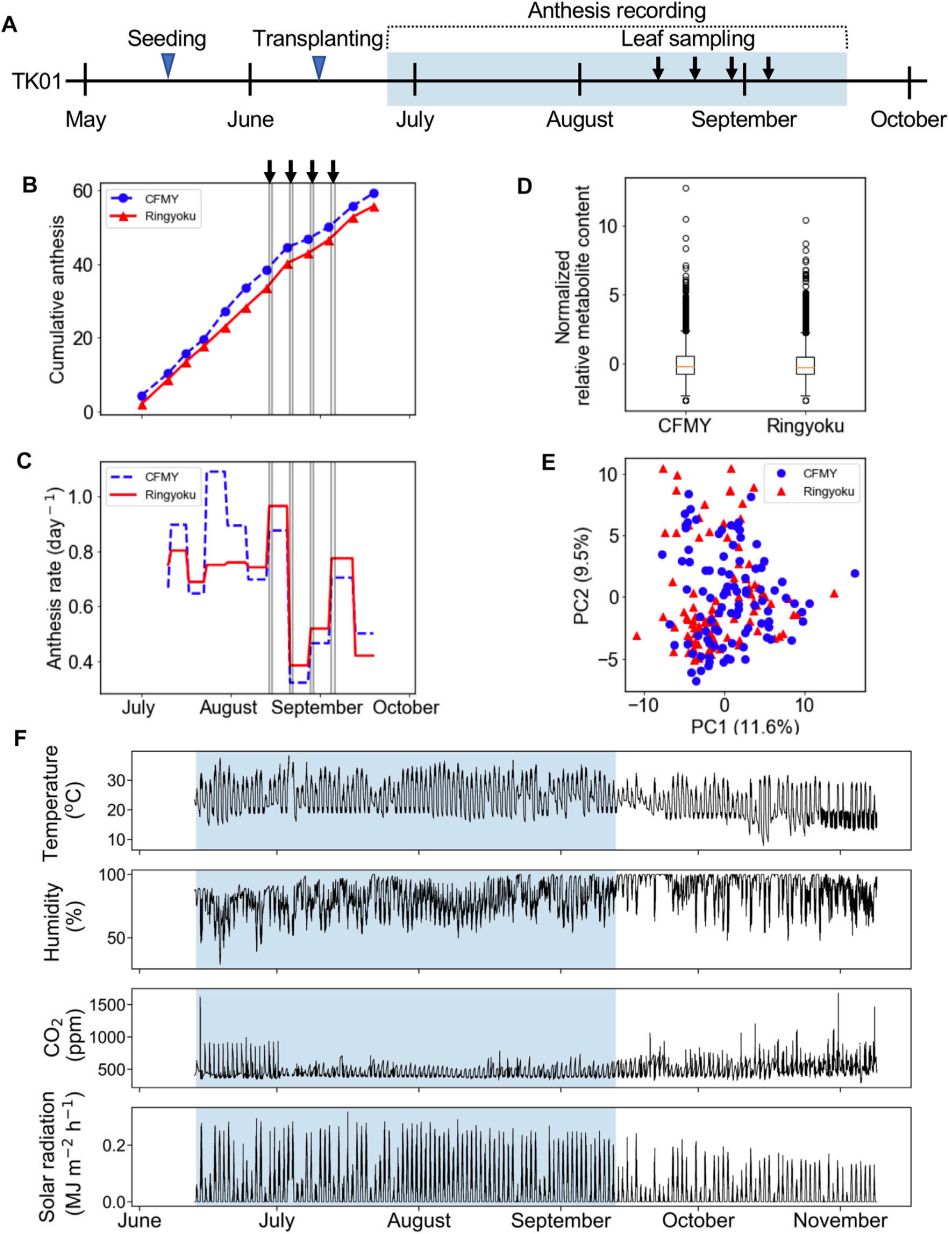
Experimental design of TK01. **(A)** Experimental timeline for leaf sampling and observation of anthesis of cultivars CFMY and Ringyoku. The blue rectangle indicates the period of the measurement of environmental factors (e.g., temperature). **(B)** Cumulative anthesis. The arrows and gray vertical lines indicate the dates of leaf sampling for metabolome analysis. **(C)** Distributions of anthesis rates were statistically the same between cultivars (Mann-Whitney U test, *p* > 0.05, CFMY; *n* = 21, Ringyoku; *n* = 21). **(D)** Box plot of the standardized relative metabolite contents of 161 metabolites in 192 leaf samples (CFMY; *n* = 96, Ringyoku; *n* = 96). **(E)** PCA score plot of the first two components (PC1 and PC2) of leaf samples (CFMY; *n* = 96, Ringyoku; *n* = 96). The metabolic profiles of the 161 metabolites were used for PCA. The numbers in parentheses in the axes are contribution ratios. **(F)** Environmental conditions measured in the experimental timeline. The environmental data in the blue background color used for LASSO analysis (E-model and C-model). The period in the blue background color is consistent with the period for leaf sampling.

In the experiment designated TK01, two tomato cultivars, Ringyoku and CFMY, were grown in Tsukuba, Japan. After transplanting the tomatoes into a greenhouse, the cumulative number of anthesis occurrences was recorded in parallel with leaflet sampling (Figure 1A). The cumulative number of anthesis occurrences was used to calculate anthesis rates (Figures 1B,C, respectively). The anthesis rates of the Ringyoku and CFMY cultivars were similar and gradually decreased over the growing period. No significant differences were observed between cultivars. During the growing period, fully developed basal and sunlit leaves were collected from plants. Leaf sampling every 2 h for 24 h was conducted four times at one-week intervals. The sampled leaves were subjected to a widely targeted metabolome analysis using a liquid chromatography-mass spectrometer. From a total of 499 targeted metabolites, 161 metabolites above the signal-to-noise ratio threshold were selected (Supplementary Table S3). The relative metabolite contents of each metabolite in all leaf samples were standardized prior to further analysis. The boxplot (Figure 1D) and PCA score plot (Figure 1E) indicated that Ringyoku and CFMY had similar metabolic profiles. Thus, we pooled the metabolic profile data obtained from the two cultivars (192 leaf samples × 161 metabolites) for further analysis. In addition, environmental data (solar irradiance, ambient temperature, relative humidity, and CO_2_ concentration) were also obtained (Figure 1F).

### 3.2 Least Absolute Shrinkage and Selection Operator Model for Anthesis Rate Prediction in TK01

We constructed three models (M-model, E-model, and C-model) to predict the anthesis rates in TK01. The model was trained and optimized to obtain predictor metabolites.

**FIGURE 2:**
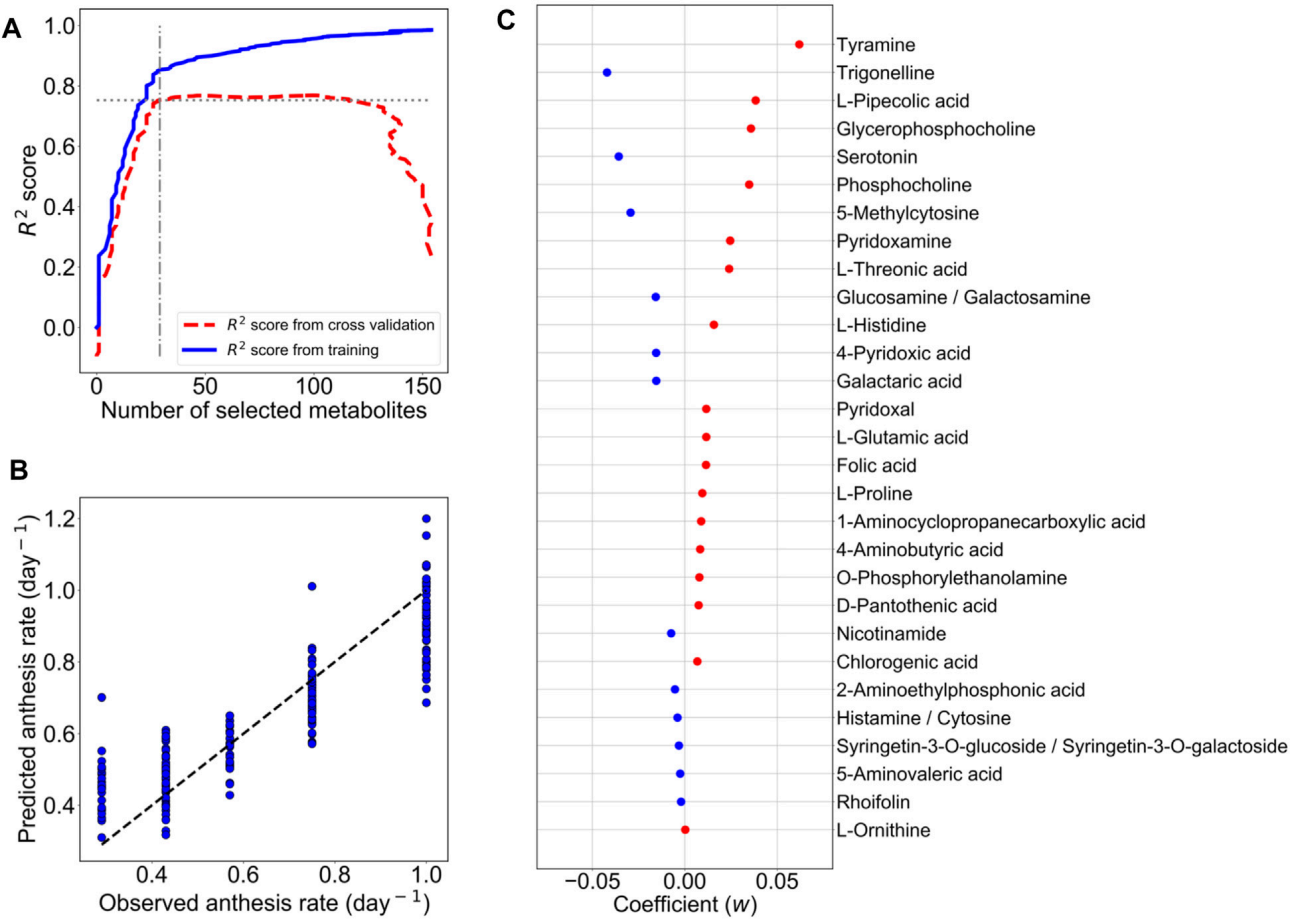
LASSO model with ten-fold cross-validation for the prediction of the anthesis rate in TK01. For LASSO regression analysis, the metabolic profiles of 161 metabolites in 192 leaf samples were employed. **(A)** The numbers of metabolites used for predictor variables versus *R* ^2^ value. The elbow point suggests the optimum set of metabolites for the prediction model. **(B)** Comparison of anthesis rates between observed and predicted values. Predicted values were obtained from the M-model with 29 selected metabolites. The dotted line represents the agreement between the observed and predicted values. **(C)** Coefficients (*w*) of 29 metabolites selected by the M-model. Red dots are positive coefficients, while blue dots are negative coefficients.

For the construction of the M-model, the metabolic profiles of 161 metabolites in 192 leaf samples were employed. During model training, we optimized the model by assigning a range of the penalty constant (α) and then measuring the prediction accuracy by cross-validation. The penalty constant (α) of the M-model was fine-tuned to optimize the best prediction model with the selected metabolites. The iteration training was performed by varied α from 5 × 10^−5^ to 0.5 (Supplementary Figure S1A). At each given α, different sets of metabolites with optimized LASSO coefficients (*w*) were selected (Supplementary Figure S1A). In each loop of a given α, the *R* ^2^ value of the M-model was calculated, and the prediction accuracy of the M-model was assessed by 10-fold cross-validation. The *R* ^2^ value and the mean squared error (MSE) of the 10-fold cross-validation were also calculated (Supplementary Figure S1B). The *R* ^2^ values of the training and cross-validation were used to determine an optimum M-model that contained the selected metabolites as the predictor metabolites for the anthesis rate (Figure 2A).

From model optimization, increasing the number of metabolites in the model increases the predictive accuracy (R^2^ values) in both training and cross-validation. Until cross-validation *R* ^2^ stopped improving while model *R* ^2^ continued to increase, this indicates overfitting in a high number of metabolites. Thus, we selected α, where the cross-validation *R* ^2^ started to plateau and was closest to training *R* ^2^ as our optimal model. In Figure 2A, the optimum number of metabolites was determined to be 29 at the elbow point on the graph that yielded the closest *R* ^2^ values between the training and cross-validation. Using the contributions of these 29 metabolites (Figure 2B) as predictor metabolites, we constructed a prediction model for TK01 (M-model). The M-model provided good prediction performance for the anthesis rates (Figure 2C). The *R* ^2^ value of the M model, *R* ^2^ s value, and MSE of the 10-fold cross-validation are summarized in Table 1.

**TABLE 1:** The prediction accuracies of the three models in TK01.

Variable used for LASSO model construction	*R* ^2^ value (LASSO model)	Cross-validation	
		*R* ^2^ value	MSE
The M-model with metabolic profiles of 29 metabolites	0.85	0.75	0.013
The E-model (only environmental factors)	0.11	0.10	0.055
The C-model with metabolic profiles of 36 metabolites and environmental factors	0.89	0.83	0.010

To examine the possibility of including environmental factors in the prediction model, we also attempted to construct a LASSO model, the E-model, using four environmental parameters (interior air temperature, interior relative humidity, interior CO_2_ concentration, and cumulative solar irradiance) recorded at 5-min intervals (Figure 1F). The prediction performance of the environmental parameters was poor (Table 1 and Supplementary Figure S2A). Finally, the C-model model was constructed using a combination of metabolites and environmental factors. The combination slightly improved the prediction accuracy of the anthesis rate (Table 1 and Supplementary Figure S2B).

### 3.3 Assessment of the Accuracy of Anthesis Rate Prediction Using the Predictor Metabolites

To assess the prediction accuracy of the anthesis rates by the contents of the 29 selected metabolites as the predictor metabolites from the M-model, datasets from two greenhouses (IA04 and IA06) were used.

#### 3.3.1 Differences in Metabolic Profiles Among Experimental Designs

**FIGURE 3:**
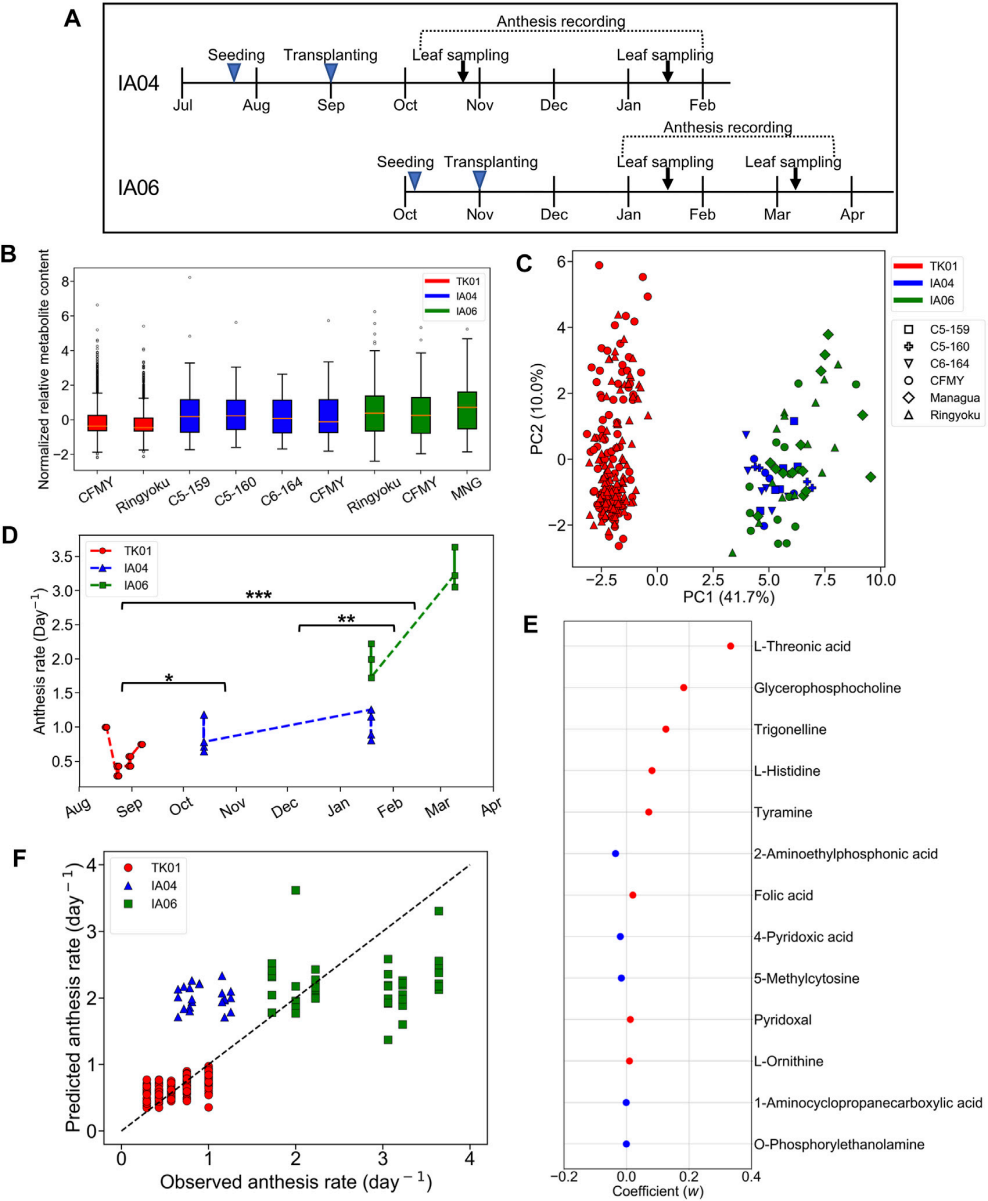
Predictability assessment of the 29 predictor metabolites with expanding metabolome datasets. **(A)** Experimental timeline for leaf sampling and anthesis measurements in IA04 (cultivars: CFMY, C5-159, and C5-16) and IA06 (cultivars: CFMY, Ringyoku, and MNG). **(B)** Box plot of standardized relative metabolite contents of the 29 predictor metabolites in each cultivar in three experimental designs (TK01, IA04, and IA06). The numbers of leaf samples (*n*): CFMY (*n* = 96) and Ringyoku (*n* = 96) in TK01, CFMY (*n* = 6), C5-159 (*n* = 6), C6-164 (*n* = 6), and C5-160 (*n* = 4) in IA04, and Ringyoku (*n* = 14), CFMY (*n* = 14), and Managua (*n* = 14) in IA06. **(C)** PCA score plot of leaf samples (*n* = 256) by using metabolic profiles of the 29 predictor metabolites from three experimental designs (TK01, IA04 and IA06). The contribution ratio is shown in parentheses for the first and second principal component (axis). The colors indicate the experimental designs, and the markers represent the cultivars. **(D)** Anthesis rates used for the LASSO model (TK01, *n* = 16; IA04, *n* = 8; IA06, *n* = 6). Asterisks indicate significant differences according to the Kruskal-Wallis test with Conover’s multiple comparison test (*, *p* < 0.05; **, *p* < 0.01; and ***, *p* < 0.001). **(E)** Model coefficients (*w*) of 13 metabolites selected in the LASSO model construction with metabolome datasets from three experimental designs (TK01, IA04 and IA06). The red dots are positive coefficients, while the blue dots are negative coefficients. **(F)** Comparison of anthesis rates between observed and predicted values obtained from the model constructed by the three datasets. The dotted line represents the agreement between the observed and predicted values.

In IA04 and IA06, the experimental designs were conducted at a different greenhouse location (Matsusaka) from TK01 (Tsukuba). In addition, these three experiments were performed in different growth seasons. Moreover, in addition to Ringyoku and CFMY, four additional cultivars were also used in IA04 and IA06 (section 2.1). During the recording of the cumulative numbers of anthesis occurrences, the leaflets were sampled for metabolome analysis at one time point around noon on 2 days (Figure 3A). Therefore, metabolic profiles must be varied by differences in the experimental designs. The relative metabolite contents of the 29 predictor metabolites on TK01, IA04, and IA06 is shown in a boxplot in Figure 3B. The distribution of the relative metabolite contents in TK01 was relatively compact, while the IA04 and IA06 data exhibited relatively larger variances. This was caused by the mixed effects of different cultivars, greenhouse conditions, and seasons. In addition, PCA for the relative metabolite contents of the 29 predictor metabolites and all leaf samples (*n* = 256) from the three greenhouses were performed to investigate the differences among the experimental designs. The TK01 leaf samples were noticeably separable from the IA04 and IA06 leaf samples, while the IA04 and IA06 leaf samples were clustered together (Figure 3C). In addition to the metabolic profiles, the anthesis rates differed among the three experimental designs (Figure 3D). The anthesis rate in IA04 was slightly higher than that in TK01, while IA06 showed the highest anthesis rate among the three experimental designs. The differences in the metabolic profiles and anthesis rate of TK01 and the two experimental designs (IA04 and IA06) made it difficult to obtain a good prediction by imputing data from IA04 and IA06 into the M-model.

#### 3.3.2 Least Absolute Shrinkage and Selection Operator Model to Assess the Prediction Accuracy of the Predictor Metabolites

We evaluated the predictive ability of 29 predictor metabolites selected from the M-model. If the predictor metabolites are biologically associated with the anthesis rate, broaden number of leaf samples from different experimental designs should provide a good prediction model. To clarify whether a more universal model could be established, the relative metabolite content of the 29 predictor metabolites and the anthesis rates obtained in TK01, IA04, and IA06 were combined and subjected to the LASSO model. A total of 13 out of the 29 metabolites that yielded the minimum MSE were selected (*R* ^2^ = 0.75) (Figure 3E). The 10-fold cross-validation results demonstrated the acceptable fitting and prediction accuracy of the model (MSE = 0.26). The model showed good prediction performance across the three datasets (cross-validated *R* ^2^ = 0.69) (Figure 3F). This result indicates that the predictor metabolites selected by the LASSO model as contributing variables in a specific dataset (TK01) could be effective for the prediction of the anthesis rate in general.

Among the two sets of metabolites selected from the M-model and this combined data model, the LASSO coefficients of the selected metabolites showed that tyramine, trigonelline, glycerophosphocholine, and L-threonic acid had a high association with the anthesis rate in both models.

### 3.4 Candidate Metabolites Associated With the Anthesis Rate

Metabolites showing significant associations with anthesis rates are attractive candidates for markers of reproductive traits, including anthesis rates, fruit development, and production. We detected candidate metabolites related to anthesis rates by LASSO analysis (Section 3.3). To understand the biological characteristics of the 29 predictor metabolites and identify candidate metabolites for future use as prediction markers, we investigated the association between the 29 selected metabolites and anthesis rates using hierarchical clustering analysis (HCL) and correspondence analysis (CA).

**FIGURE 4:**
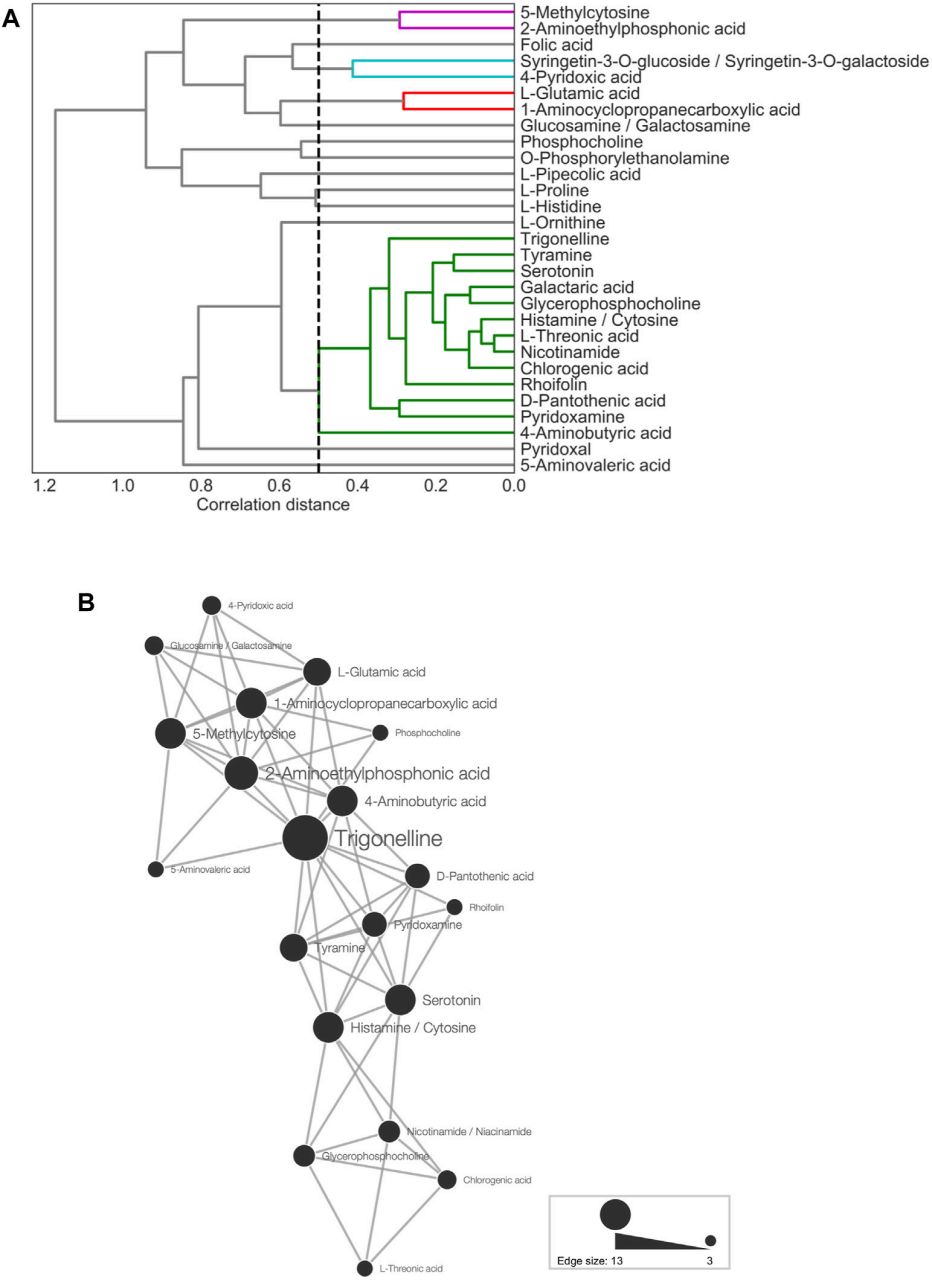
Metabolite association of 29 predictor metabolites. **(A)** Dendrogram representing agglomerative clustering of the correlation distances of the 29 selected metabolites in average linkage. The cluster threshold was 0.5, as indicated by the black dotted line. Lines of the same color represent the same clusters. **(B)** Network for metabolites (threshold: ≤15th percentile of Euclidean distances). The node size represents the number of edges linked to other metabolites.

First, HCL was used to visualize the metabolic profiles of TK01, IA04, and IA06. Pearson correlation coefficients (*r*) between each pair of the 29 predictor metabolites were obtained to evaluate the similarity in the profiles (Supplementary Figure S3). Strong correlations were observed, particularly in the top selected metabolites, such as tyramine, trigoneline, glycerophosphocholine, and serotonin, of the M-model (Figure 4A). This result suggests that each of these metabolites plays a similar and important role in anthesis rate estimation. It indicates that it is possible to choose only a small number of metabolites as key predictors of anthesis rates.

Next, CA was conducted for network analysis to elucidate the associations among the 29 predictor metabolites. In the metabolic network (Figure 4B), all of the connected metabolites were amines, except for chlorogenic acid, rhoifolin, and L-threonic acid. Thus, the nitrogen-containing metabolites showed similar accumulation patterns across the leaf samples (Figure 4B). Among all metabolite-to-metabolite edges, trigonelline has the most edges linked to other metabolites, indicating that trigonelline is a major coexisting metabolite with others.

**FIGURE 5:**
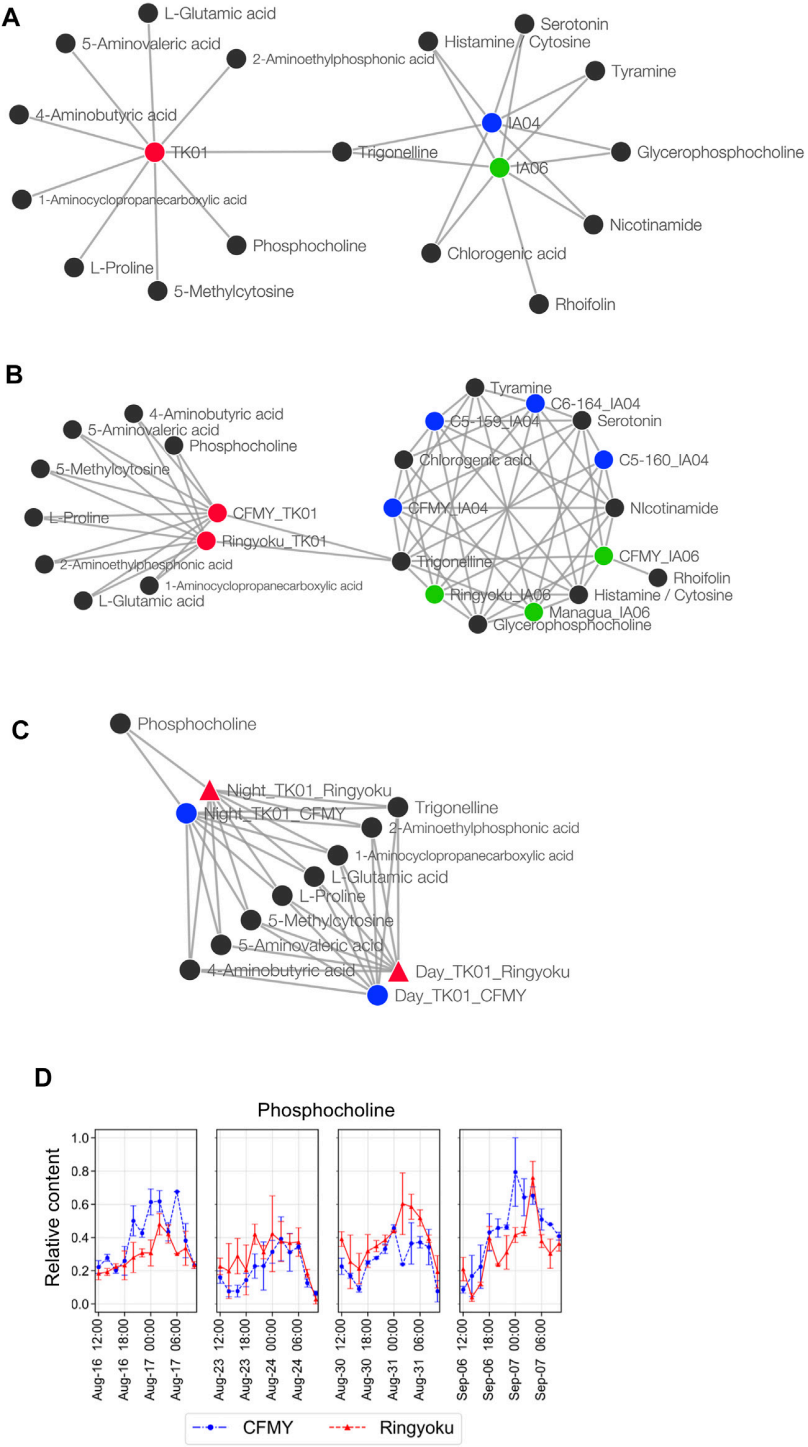
Metabolite association with experimental designs, cultivars, and sampling times. **(A)** Network of metabolites and growth conditions. **(B)** Network of metabolites and cultivars. The cultivars were divided into subcategories of experimental design; for example, the CFMY samples were divided into three and labeled CFMY_TK01, CFMY_IA04, and CFMY_IA06. **(C)** Network of metabolites and sampling times. **(D)** Diurnal changes of the relative content of phosphocholine (scaled between 0 and 1).

CA was also conducted for network analysis to elucidate the associations between the 29 predictor metabolites and leaf sample characteristics, that is, experimental designs, cultivars, and sampling times. Among the leaf sample characteristics, the experimental design was the only factor displaying a clear separation in PCA (Figure 3C), whereas the cultivars and sampling times did not show distinct separation (Supplementary Figures S4B,C). Thus, in CA, we first examined the network between the predictor metabolites and the experimental designs (TK01, IA04, and IA06). In the network (Figure 5A), IA04 and IA06 shared seven similarly dominant metabolites. Four out of the seven metabolites, glycerophosphocholine, serotonin, trigonelline, and tyramine, were in the top five of the 29 predictor metabolites (Figure 2C). TK01 had nine highly associated metabolites. Among them, one metabolite, trigonelline, was linked to all three experimental designs in the network (Figure 5A). Next, we examined the association between metabolites and cultivars. In the metabolite to cultivar network (Figure 5B), a network pattern similar to the experimental design was observed. The cultivars IA04 and IA06 shared highly associated metabolites but did not share with the cultivars in TK01, except trigonelline, which was associated with all cultivars (Figure 5B).

### 3.5 Candidates of Stable Metabolites for the Prediction of the Anthesis Rate

Taking into account the leaf sampling time, metabolite content generally changes according to the circadian rhythm. For future use as key indicators of anthesis rate, metabolites whose contents do not change depending on the leaf sampling time are preferred. Because the leaf samples from TK01 were collected every 2 h for a day in time-series format, we constructed a Euclidean distance network of TK01 samples to identify the metabolite associated with leaf sampling time, namely day (06:00‒18:00) or night (20:00‒04:00) (Figure 5C). Among the nine metabolites strongly associated with TK01, phosphocholine was highly associated only at night. This result is consistent with the accumulation pattern of phosphocholine, which showed a diurnal bell-shaped pattern peaking at night (Figure 5D). Eight other metabolites, including trigonelline, shared associations during both day and night, indicating high metabolite production, which may produce stable production throughout the day.

**FIGURE 6:**
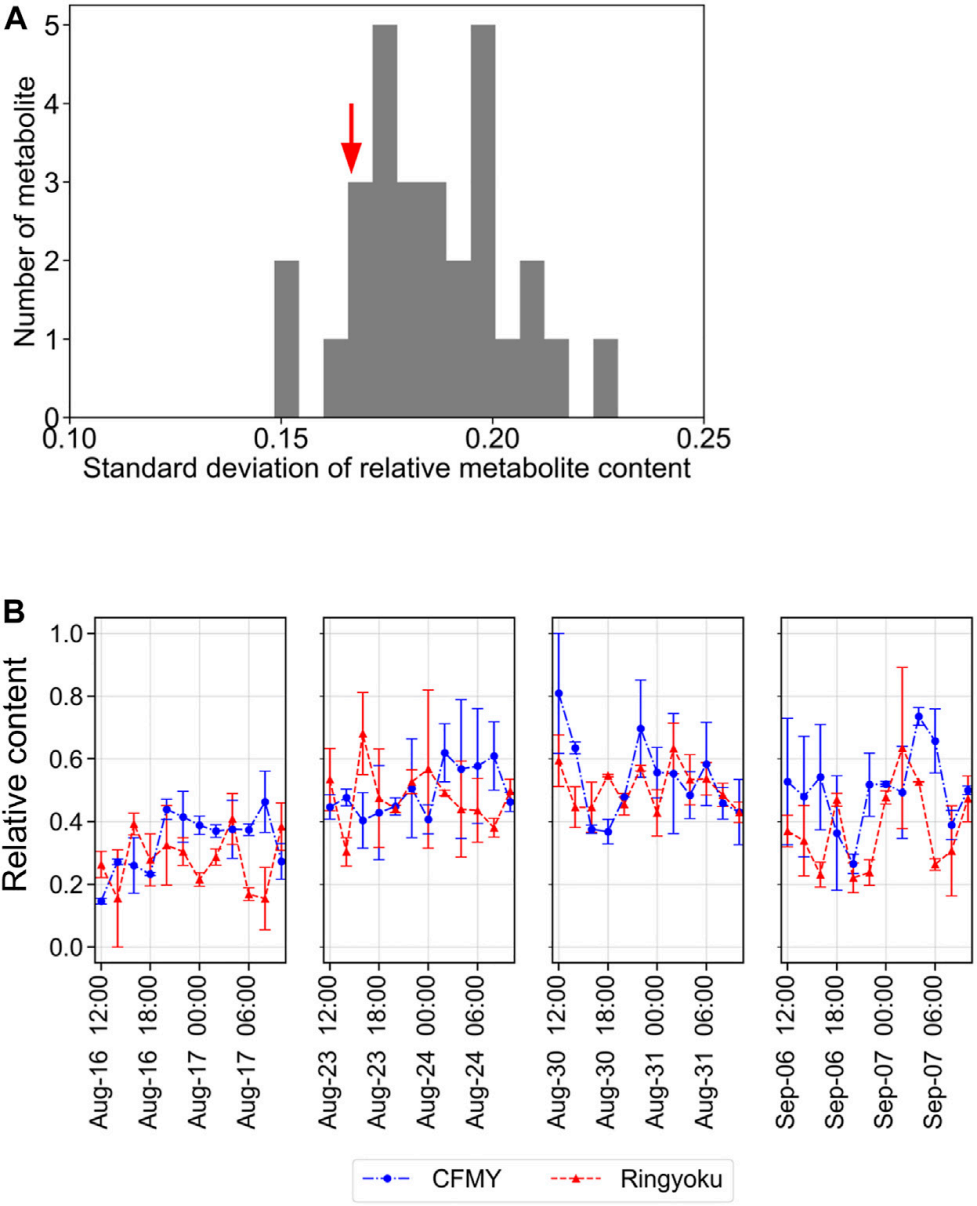
Diurnal fluctuations of metabolite content in tomato leaves. **(A)** Distribution of the standard deviations of 29 metabolites. The red arrow indicates the standard deviation of trigonelline at 0.167. **(B)** Diurnal fluctuations of the relative content of trigonelline (scaled between 0 and 1).

To further evaluate the diurnal fluctuations of the 29 LASSO-selected metabolites in TK01, the relative contents of each metabolite were scaled between 0 and 1. The distribution of the standard deviations (SD) of the 29 metabolites is shown in Figure 6A. The standard deviations of the metabolite contents ranged from 0.148 to 0.230. Among these, the standard deviation of trigonelline was relatively small (SD = 0.167). In addition, the trigonelline content was relatively stable over the course of a day (Figure 6B) compared to that of the other metabolites, such as phosphocholine, glycerophosphocholine, L-glutamic acid, and 4-aminobutyric acid, which exhibited strong diurnal fluctuations (Supplementary Figure S5).

Taken together, our results suggest that trigonelline is an attractive metabolite for use as a marker of the anthesis rate of tomatoes. Trigonelline was one of the top five LASSO-selected metabolites for the prediction of the anthesis rate (Figures 2C, 3E), showed no diurnal changes, and exhibited stable content among the different cultivation conditions and varieties (Figures 6A,B). Other metabolites among the top five, such as tyramine, were also available not only for the prediction of the anthesis rate, but also as markers under specific cultivation conditions.

## 4 Discussion

Machine learning approaches have the potential to provide prediction models for agricultural traits and effectively identify metabolites, genes, or environmental factors associated with these traits (Menéndez et al., 2011; Acharjee, 2013; Das et al., 2018; Du et al., 2019; Sawada et al., 2019). Our study employed LASSO regularized linear regression model analysis to construct a prediction model of the anthesis rate using leaf metabolome data as predictor variables and identify the 29 predictor metabolites as candidate biomarkers. Importantly, we identified trigonelline as a key metabolite for anthesis rate prediction using the LASSO models and CA. Moreover, because the trigonelline content in the leaf was relatively stable over the course of a day, it was identified as an attractive biomarker of anthesis rate.

### 4.1 Possible Uses of Least Absolute Shrinkage and Selection Operator-Selected Metabolites as Biomarkers

The prediction of reproduction and fruit development in tomato is a powerful tool for the diagnosis of plants and the optimal management of the environmental conditions to maximize plant yields. Since anthesis is directly linked to tomato fruit production, it is a good index with which to evaluate tomato cultivation. The identification of metabolites involved in anthesis can be employed as metabolite markers for the prediction of anthesis and yield.

In the construction of the models using LASSO, unimportant metabolites were penalized by L1 regularization, leaving more prominent metabolites after variable selection. A reduction in the number of metabolites is desirable, because a smaller number of metabolites can be more easily measured for future use as biomarkers. As a result, 29 metabolites, including both primary and specialized (secondary) metabolites, were selected from among 161 metabolites. Most of the 29 selected metabolites were nitrogen-containing compounds, such as amino acids and their derivatives, alkaloids, amines, and phospholipids. The LASSO-selected metabolites could indicate the nitrogen status associated with the anthesis rate in tomatoes.

Among the 29 metabolites, trigonelline (*N*-methylnicotinate), a quaternary ammonium, exhibited a metabolic profile similar to that of the majority of the selected metabolites. (Figure 4B). In addition, trigonelline demonstrated the greatest association with all three growth conditions and all cultivars, while other metabolites were associated with only leaf samples from particular experiments (Figures 5A–C). Moreover, compared to other metabolites, trigonelline showed a relatively stable accumulation over the course of a day (Figures 5D, 6B and Supplementary Figure S5). Among 29 metabolites associated with anthesis rate, trigonelline was shown to be a key metabolite related to anthesis rate. These results support that trigonelline is a suitable biomarker without diurnal fluctuations.

Trigonelline is known to increase in tomato leaves in response to increased nitrogen content in nutrient solutions (Tyihák et al., 1988), and can thus serve as a possible indicator of nitrogen status within the plant body. Therefore, we investigated the correlation between trigonelline content in leaves and nitrogen fertilizer absorption in IA04 and IA06 (Supplementary Table S9). The results showed a positive correlation (*r* = 0.56, *p* < 0.05) in IA06 and a weak correlation (*r* = 0.30, *p* < 0.05) in IA04, supporting this hypothesis. Trigonelline is synthesized from nicotinic acid, which is a metabolite of the nicotinamide adenine dinucleotide (NAD) synthesis/degradation (Ashihara, 2006). The functions of trigonelline in plants have been reported in terms of various aspects, such as cell cycle regulation, nodulation, and reduction of oxidative stress (Minorsky, 2002). A recent study reported on the function of trigonelline as a detoxified metabolite of excess nicotinic acid in the NAD cycle (Li et al., 2017). The demethylation of trigonelline regenerated nicotinic acid for utilization in NAD synthesis as a reservoir metabolite. Demethylating activity has also been observed in the leaves of some plants, as well as in coffee plant seeds, during germination (Ashihara, 2006). In *Arabidopsis thaliana*, NAD is known to play an important role in growth phase transition (Hashida et al., 2016). In a previous study, the perturbation of NAD redox homeostasis due to the overexpression of genes involved in NAD synthesis resulted in the ectopic generation of reactive oxygen species, leading to early flower stalk wilting and shortened plant longevity (Hashida et al., 2016). In addition, NAD accumulation was reported in pollen before germination, indicating that NAD metabolism plays a crucial role in pollen maturation (Hashida et al., 2013). Our hypothesis is that trigonelline may be involved in flower development via NAD homeostasis, however, further experiments are required to confirm this hypothesis.

### 4.2 Improving Predictability by Using Environmental Data

Although we attempted to use environmental factors to predict reproductive traits, the prediction performances of the generated models were poor (Table 1 and Supplementary Figure S2A). These results support our understanding that short-term environmental data are insufficient for yield prediction. Accumulated historic datasets of environmental factors may be required to achieve more accurate predictions (Adams, 2002; Qaddoum et al., 2013; Saito et al., 2020). On the other hand, the combination of metabolome and environmental factor data resulted in improved prediction performance (Table 1). Considering a plant as an autotrophic production system, it is reasonable that a combination of environmental factors (system inputs) and metabolic status (a system internal condition) can produce more accurate production estimates (system outputs) than either one individually. Thus, monitoring both types of factors in a greenhouse system management is likely to yield the best prediction performance.

### 4.3 Machine Learning Algorithms for Metabolome Data

Among the machine learning approaches, LASSO linear regression analysis was chosen for the following reasons. First, linear regression is often used to estimate biological rates (Schneider et al., 2010). Thus, linear regression seems to be an appropriate choice for our experiments. Second, our dataset contained more variables than samples, which could lead to severe overfitting in a more complex model (Trunk, 1979). A simpler model, such as a linear regression model combined with LASSO regularization, is preferred; therefore, the LASSO linear regression method is employed in this study. In fact, we have previously tested several other regression algorithms, including ridge regression, random forest regressor, k-nearest neighbor regression, and support vector regression (Pedregosa et al., 2011; VanderPlas, 2016), all of which performed worse than or the same as the LASSO model with our dataset (data not shown). A detailed comparison of these algorithms will be described elsewhere. Based on this knowledge, LASSO was chosen for this study.

## Data Availability

The datasets presented in this study can be found in online repositories. The names of the repository/repositories and accession number(s) can be found below: http://prime.psc.riken.jp/archives/data/DropMet/059/.
