# Supplementary material for: Assessment of Greenhouse Tomato Anthesis Rate Through Metabolomics Using LASSO Regularized Linear Regression Model
Source: Front Mol Biosci. 2022 Mar 1;9:839051. doi: 10.3389/fmolb.2022.839051 (PMC8923526; doi:10.3389/fmolb.2022.839051)
Supplement: Supplementary file 1 [file Presentation1.pdf]

Supplementary Material

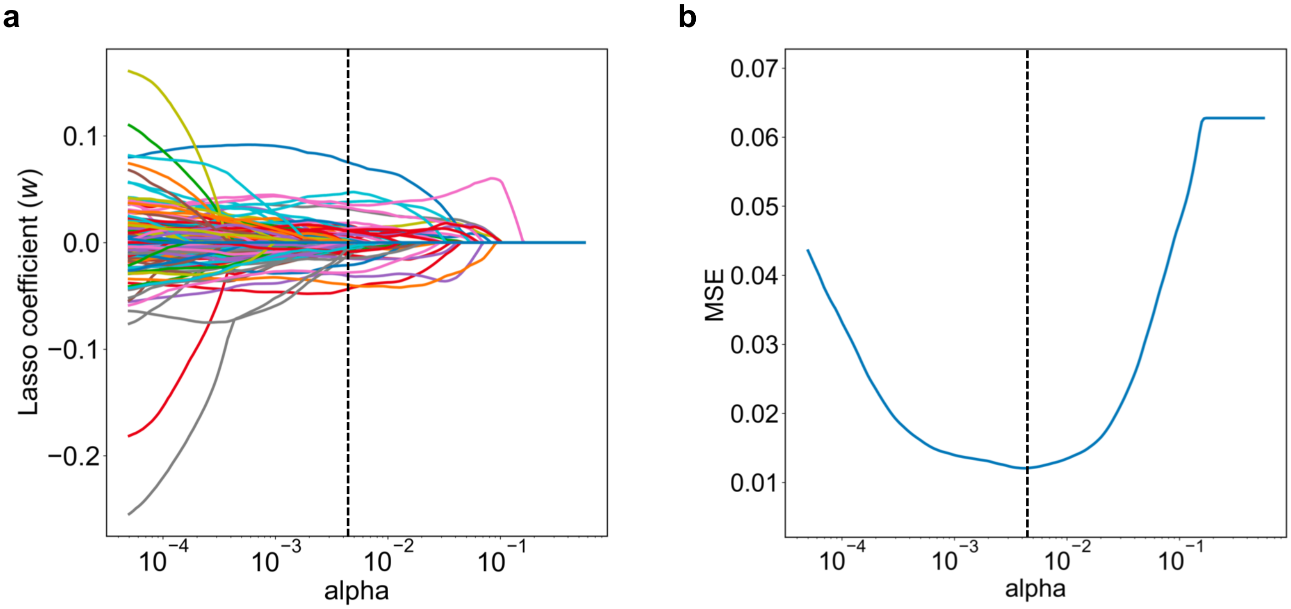

Figure S1. Construction of the M-model in TK01. A prediction model was constructed from metabolic profiles of 161 metabolites. (a) Model coefficients (*w*) for 161 metabolites. The coefficients were determined based on L1 regularization of α (under 5×10^{-5} to 0.56). Each line represents the model coefficient of a metabolite. The black dotted line displays the value of α minimizing the objective function. (b) MSE values for α ranging from 5×10^{-5} to 0.56. The black dotted line displays the value of α minimizing the objective function.

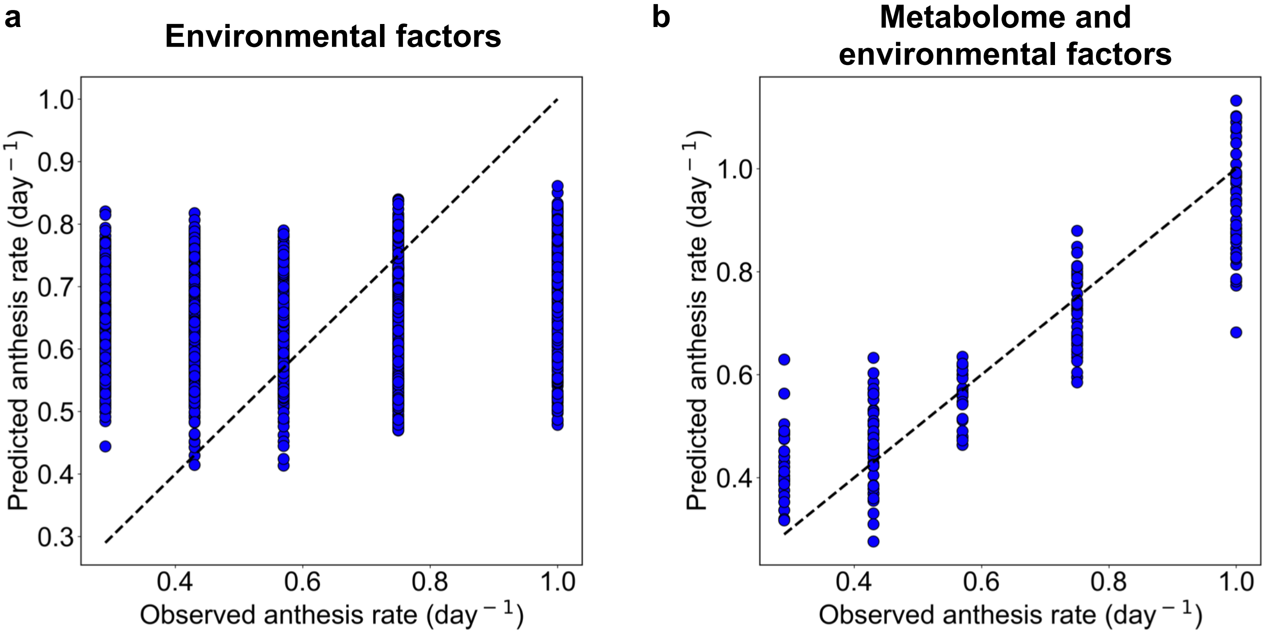

Figure S2. Prediction accuracy of anthesis rates in the E-model and C-model. Comparison between prediction and observed values. The dotted line represents the agreement between the predicted and observed values. (a) E-model constructed using only environmental data. (b) C-model constructed with a combination of metabolome and environmental data.

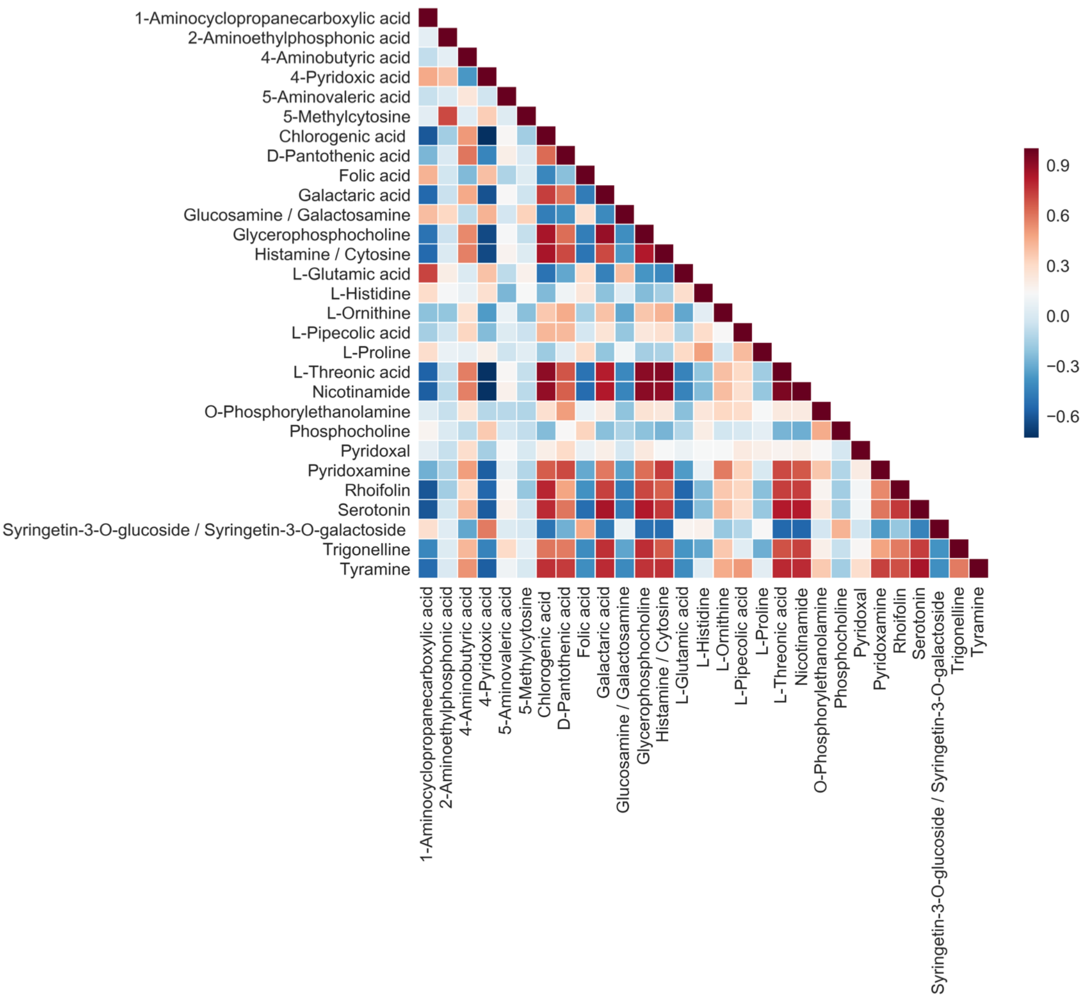

Figure S3. Pearson correlation coefficients of the 29 selected metabolites. A heatmap representing the Pearson correlation coefficients (*r*) of each pair from the 29 selected metabolites.

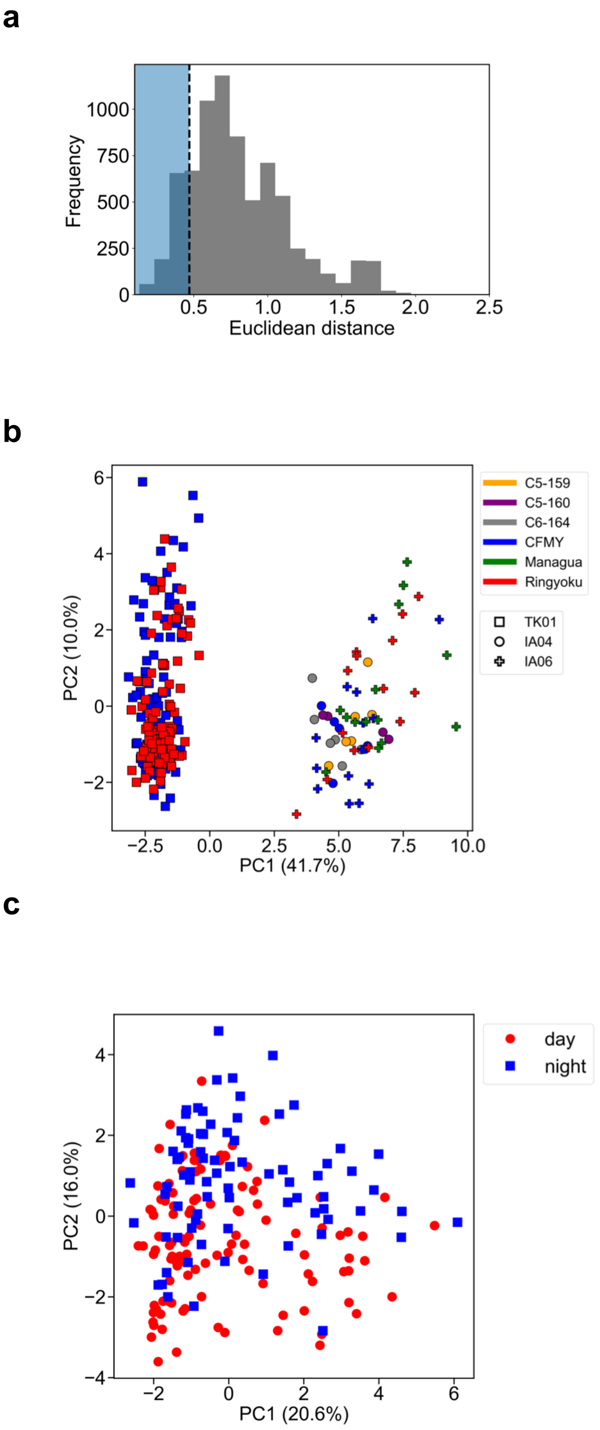

Figure S4. CA and PCA. (a) Frequencies of Euclidean distances between the metabolites and leaf samples. The blue rectangles indicate the significant range of distances (≤15th percentile) used to construct the networks. (b) A PCA score plot of leaf samples (n = 256) for experimental designs and cultivars. The colors represent the cultivars. Markers indicate the experimental designs. (c) PCA score plot of leaf samples (n = 192) in TK01 for the sampling time of day (06:00–18:00) and night (20:00–04:00).

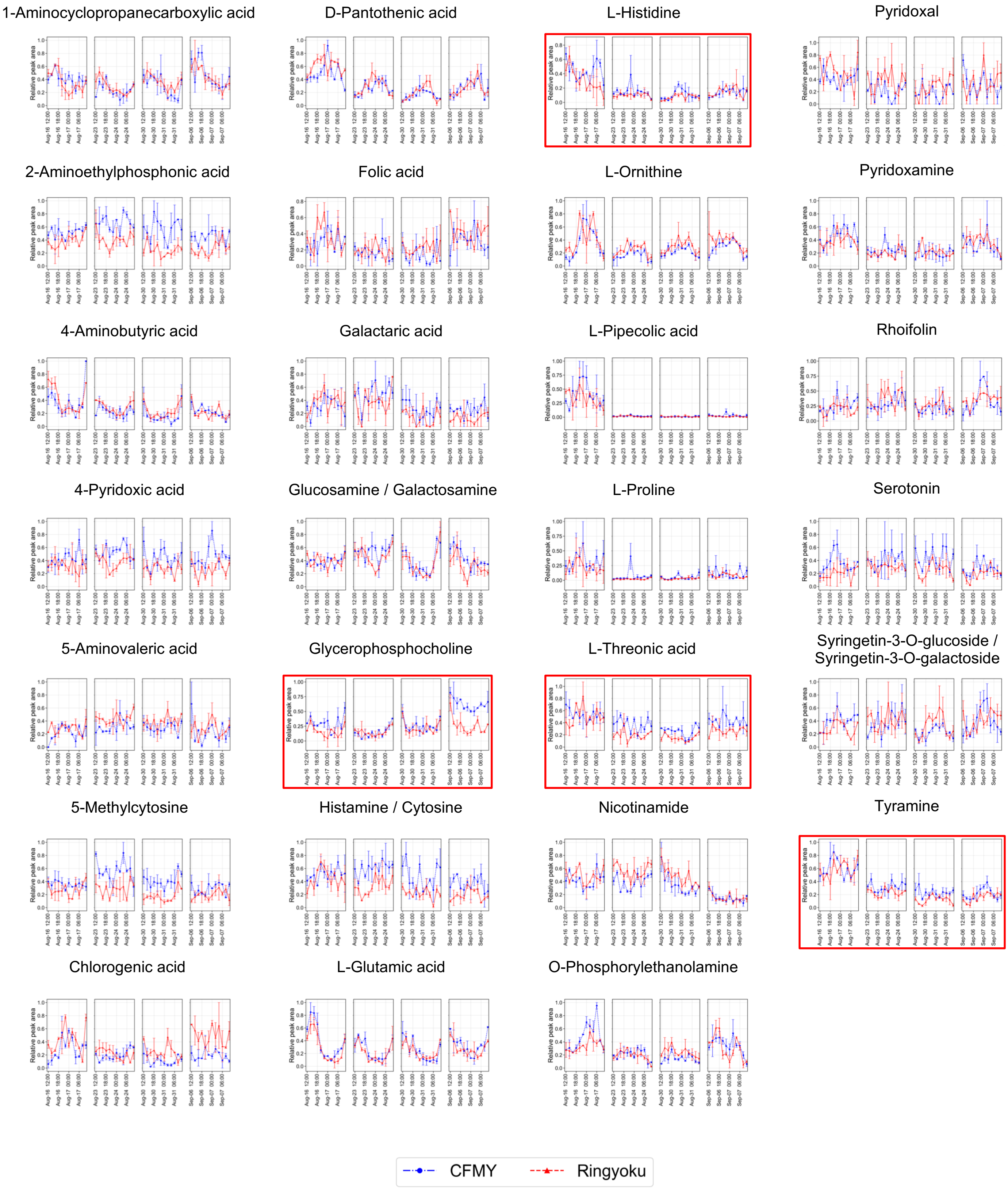

Figure S5. Diurnal fluctuations of the relative contents of the LASSO-selected metabolites. The top five of metabolites that were selected from the model constructed based on the three datasets are highlight in red rectangles.
